# Physical synthesis methodology and enhanced gas sensing and photoelectrochemical performance of 1D serrated zinc oxide–zinc ferrite nanocomposites

**DOI:** 10.1186/s11671-015-1059-0

**Published:** 2015-09-03

**Authors:** Yuan-Chang Liang, Shang-Luen Liu, Hao-Yuan Hsia

**Affiliations:** Institute of Materials Engineering, National Taiwan Ocean University, Keelung, 20224 Taiwan

**Keywords:** Nanocomposite, Oxides, Crystallites, Morphology, Sensing performance

## Abstract

We successfully prepared one-dimensional ZnO–ZnFe_2_O_4_ (ZFO) heterostructures for acetone gas-sensing and photoelectrochemical applications, by using sputter deposition of ZFO crystallites on ZnO nanostructure templates. The nanoscale ZFO crystallites were homogeneously coated on the surfaces of the ZnO nanostructures. Electron microscope images revealed that the ZnO–ZFO heterostructures exhibited a serrated surface morphology. Coating the ZnO nanostructures with a ZFO aggregated layer appreciably enhanced their acetone gas-sensing capability at 250 °C in comparison with pure ZnO nanostructures. The presence of many depleted nanoscale ZFO crystallites, the rugged surface of the heterostructures, and electron depletion at the ZnO/ZFO interface might contribute to the enhanced acetone gas-sensing response. Furthermore, the larger surface area and higher light absorption of ZnO–ZFO relative to the surface area and light absorption of ZnO were correlated with a substantial enhancement of the photocurrent value of ZnO–ZFO in photoelectrochemical tests produced by the simulated solar light irradiation.

## Background

Spinel oxides have a wide range of technical applications and are described as AB_2_O_4_. In these oxides, the anions (O^2−^ ions) form a face-centered, cubic, and close-packed structure, and cations occupy four-coordinated and six-coordinated sites [1, 2]. ZnFe_2_O_4_ (ZFO) is a promising spinel oxide that has fascinating electrochemical, optical, and magnetic properties [3, 4]. This ferrite compound shows visible reducing gas-sensing properties [5]. Moreover, ZFO has a bandgap in the visible light wavelength range and is widely used for photocatalytically degrading pollutants [6]. Various methods, including the aspartic-acid-assisted combustion method and sputtering techniques, are used to synthesize ZFO in powder and thin-film forms for scientific applications [1, 3]. Because of its high surface-to-volume ratio, ZFO demonstrates unique physical and chemical properties. These functions have motivated recent studies on the synthesis and characterization of one-dimensional (1D) ZFO. For example, carbon-decorated ZFO nanowires have been prepared through the calcination of glucose-coated ZnFe_2_(C_2_O_4_)_3_ nanowires synthesized from glucose-containing microemulsion solutions, and used as a highly reversible lithium-ion anode material [7]. Preferentially oriented ZFO nanowire arrays were fabricated through the postannealing of ZnFe_2_ nanowires, and the magnetic properties of the as-synthesized ZFO were investigated [4]. Floriated ZFO with porous nanorod structures was synthesized using a hydrothermal method, and its use as a photocatalyst in hydrogen production under visible light was investigated [8]. Highly ordered ZFO nanotube arrays were prepared using a sol–gel AAO template method, and they were found to display high sensitivity to organic gases [9]. Although several chemical methodologies for preparing ternary ZFO nanostructures have been demonstrated, a mature physical method for synthesizing 1D ZFO remains technologically challenging because of the complex composition of this compound.

Several 1D hybrids or heterostructures consisting of binary semiconductors have been shown to enhance gas-sensing properties and demonstrate higher photoelectrochemical performance compared with their single counterpart [10, 11]. However, in contrast to the numerous studies on binary semiconductor hybrids, reports on 1D hybrids or heterostructures integrated with ternary spinel semiconductor compounds are considerably low in number [12, 13]. In this study, ZFO crystallites with high crystal quality were prepared through radio-frequency (RF) sputtering. They were homogeneously decorated on ZnO nanowires to form 1D ZnO–ZFO heterostructures. In a previous study, spinel ZFO-nanoparticle-coated rod-like ZnO nanostructures were successfully synthesized using a low-temperature hydrothermal strategy, and they exhibited high selectivity for *n*-butanol [14]. In another study, efficient visible-light photoelectrochemical oxidation of water was realized in nanostructured ZnO–ZFO heterojunctions [15]. According to relevant studies, a 1D hybrid obtained by combining ZnO and ZFO has potential applications in gas detection and high-efficiency photoelectrochemical sensing devices because of the large difference between bandgaps. However, the morphology and crystal quality of hybrids or heterostructures are known to affect their sensing performance [16, 17]. Moreover, for a given material system, different preparation methodologies yield nanostructures that exhibit different crystal features. Understanding the correlation between microstructure and sensing performance is crucial for designing hybrids or heterostructures that demonstrate the required device performance. In the current study, serrated ZFO-crystallite-decorated ZnO nanostructures were synthesized using physical methodologies. Subsequently, the gas and photoelectrochemical sensing performance of sensors fabricated from 1D ZnO–ZFO heterostructures was correlated with that of their microstructures to examine the possible use of this heterostructure in small sensing devices.

## Methods

In this study, ZFO crystallites were fabricated using RF magnetron sputtering in an Ar/O_2_ (Ar:O_2_ = 3:1) mixed ambient. The growth temperature of the ZFO was maintained at 350 °C. The gas pressure during deposition was fixed at 20 mTorr and sputtering power was fixed at 80 W. Cross-linked ZnO nanowires were employed as templates to fabricated ZFO crystallite-coated ZnO nanostructures. ZFO nanofilms with tens of nanometers were grown onto ZnO nanowire templates according to the aforementioned thin-film deposition parameters to form ZnO–ZFO heterostructures. The cross-linked ZnO nanowires were synthesized through thermal vapor evaporation in a horizontal quartz tube furnace and detailed experimental setup was described elsewhere [18]. Sample crystal structures were investigated by X-ray diffraction (XRD; Panalytical X’Pert Pro MPD) using Cu Kα radiation. The surface morphology of the samples was investigated by scanning electron microscopy (SEM; Hitachi S-4800). The detailed microstructures of the as-synthesized samples were characterized by high-resolution transmission electron microscopy (HRTEM; Philips Tecnai F20 G2). Silver glue was used to fabricate two metal electrodes onto the samples for electric measurements. To measure acetone gas-sensing properties, samples were placed in a closed vacuum chamber, and various concentrations (50–750 ppm) of acetone gas were introduced into the chamber, using dry air as the carrier gas. The sensor response to acetone gas is defined as the ratio (*R*a/*R*g). *R*a is the electrical resistance of the sensor in the absence of acetone gas. *R*g is the electrical resistance of the sensor in acetone gas. The photoelectrochemical (PEC) properties were measured in a convenient three electrodes electrochemical system (SP-50 Potentiostat/Galvanostat). Na_2_SO_4_ aqueous solution (0.1 M) was used as electrolyte. Work electrodes were made of ZnO nanowires and ZnO–ZFO heterostructures on conductive F-doped SnO_2_ glasses. Ag/AgCl (1 M KCl) electrode was used as a reference electrode, and a platinum wire was used as a counter electrode. A 100 W Xe arc lamp was used as the illumination source for photocurrent measurement.

## Results and Discussion

**Fig. 1:**
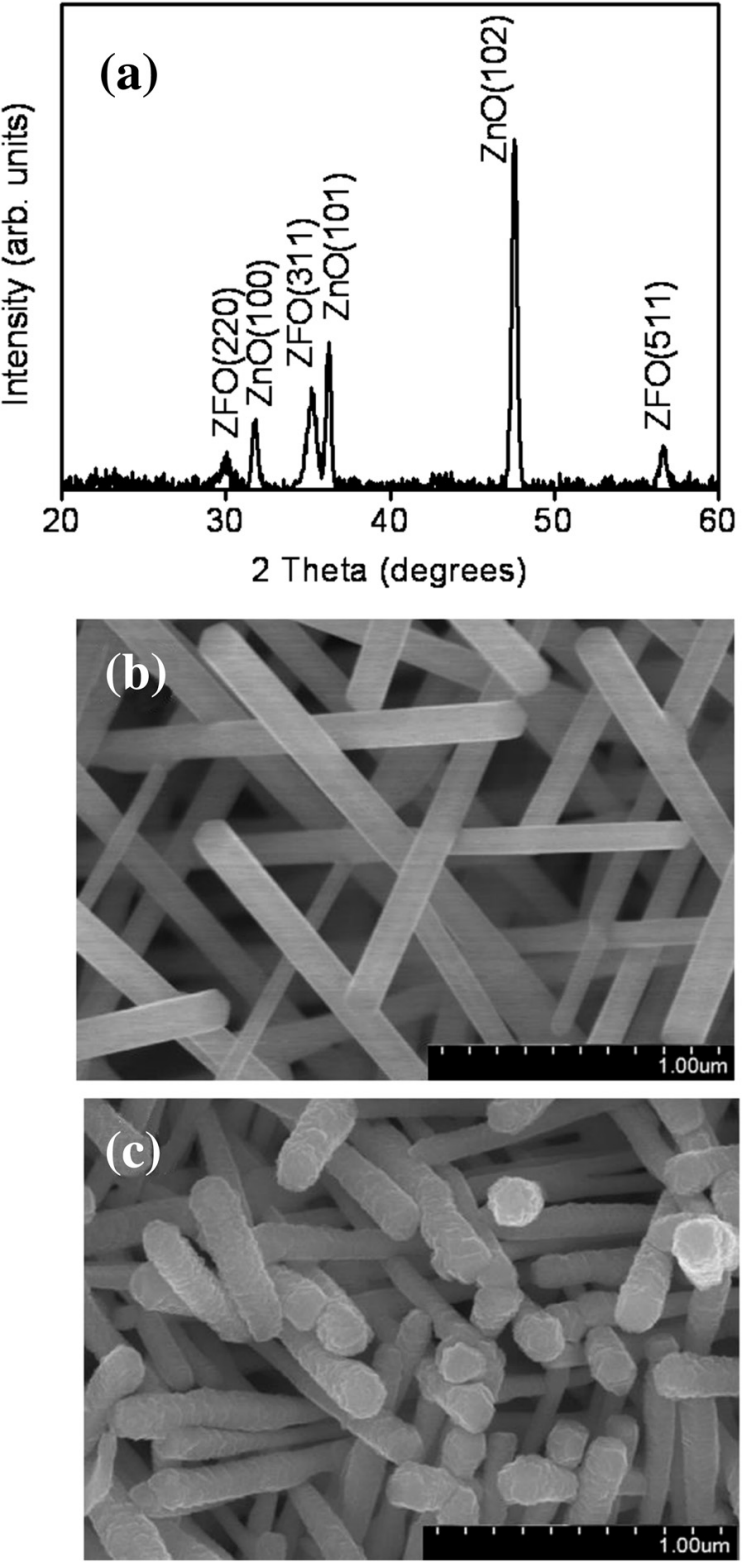
XRD and SEM analyses of the ZnO–ZFO heterostructures: **a** XRD pattern of the ZnO–ZFO nanostructures. **b** SEM image of the as-synthesized ZnO nanowires. **c** SEM image of the ZnO–ZFO core-shell heterostructures

Figure 1a shows the XRD pattern of cross-linked ZnO nanostructures coated with ZFO nanofilms. Several intense ZnO Bragg reflections were observed, and we assigned them to the (100), (101), and (102) planes according to JCPDS No. 36-1415. The intense Bragg reflections originated from nonpolar crystallographic planes and revealed the cross-linked feature of the hexagonal ZnO nanostructures. Moreover, several clear, distinct Bragg reflections of ZFO were observed in the XRD pattern. The Bragg reflections of (220), (311), and (511) at approximately 30°, 35.2°, and 56° corresponded to cubic ZFO with a spinel structure, respectively (JCPDS No. 22-1012). No other apparent reflections originating from impurity phases were detected. The XRD results showed highly crystalline ZFO crystals covering the cross-linked ZnO nanostructures, suggesting that the ZnO–ZFO heterostructures have high crystalline quality. Figure 1b shows the morphology of the ZnO nanostructures. The surface of ZnO is smooth. An SEM image of ZnO nanostructures coated with ZFO nanofilms is shown in Fig. 1c. The straight feature of the ZnO nanostructures was maintained after coating them with ZFO nanofilms. In comparison with the SEM micrograph of pure ZnO, the ZFO crystallites formed an aggregate layer on the ZnO nanostructures, resulting in the ZnO–ZFO nanostructure surface being rugged.

**Fig. 2:**
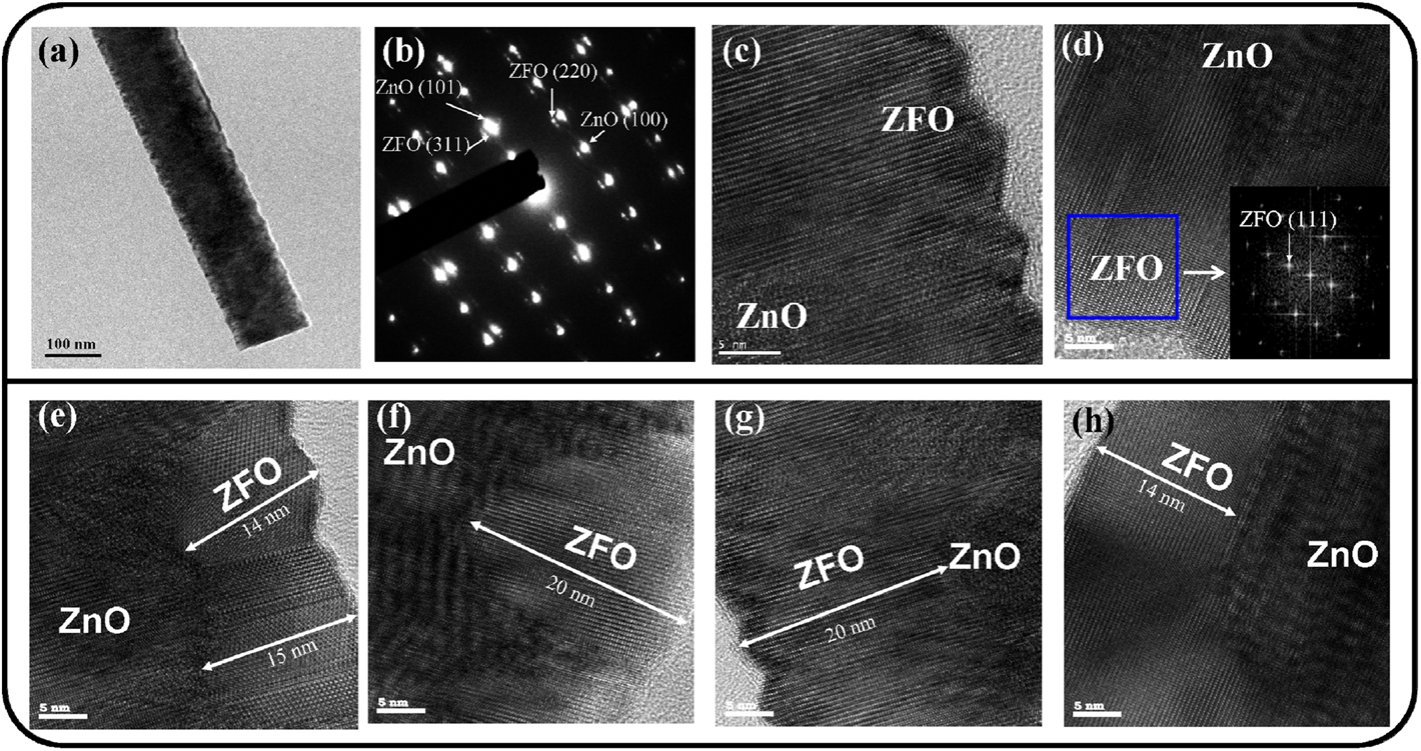
TEM analyses of the ZnO–ZFO heterostructures: **a** low-magnification TEM image of a ZnO–ZFO heterostructure. **b** The SAED pattern of the heterostructure. **c**–**d** HRTEM images taken from the local regions of the heterostructure. **e**–**h** Micrographs exhibit the thickness of the ZFO crystallites

Figure 2a shows a low-magnification TEM image of a single ZnO–ZFO nanostructure, showing the rugged surface of the nanostructure. Figure 2b shows the selected area electron diffraction (SAED) pattern of the nanostructure. The SAED pattern shows ordered, sharp, and bright spots of various sizes, revealing a high-quality crystalline ZnO–ZFO nanostructure. The clear spots originated from the wurtzite ZnO core, and the other relatively tiny spots originated from the cubic ZFO crystallites. Figure 2c, d shows high-resolution TEM (HRTEM) images of various regions of the nanostructure. They reveal that ZFO crystallites attached to ZnO caused the nanoscale surface ruggedness of ZnO–ZFO. The clear lattice fringes in the regions occupied by ZFO crystallites reveal long-range ordered atomic arrangements in the crystals. The corresponding fast Fourier transform (FFT) pattern of the ZFO crystallite is also shown in the inset of Fig. 2d. The FFT pattern shows the crystal orientation of the single crystalline ZFO. The TEM analysis showed that highly crystalline ZFO crystallites were attached to the ZnO nanostructure surface, forming ZnO–ZFO heterostructures. The ZFO crystallites had thicknesses in the approximate range of 10–20 nm, as exhibited in Fig. 2e-h).

**Fig. 3:**
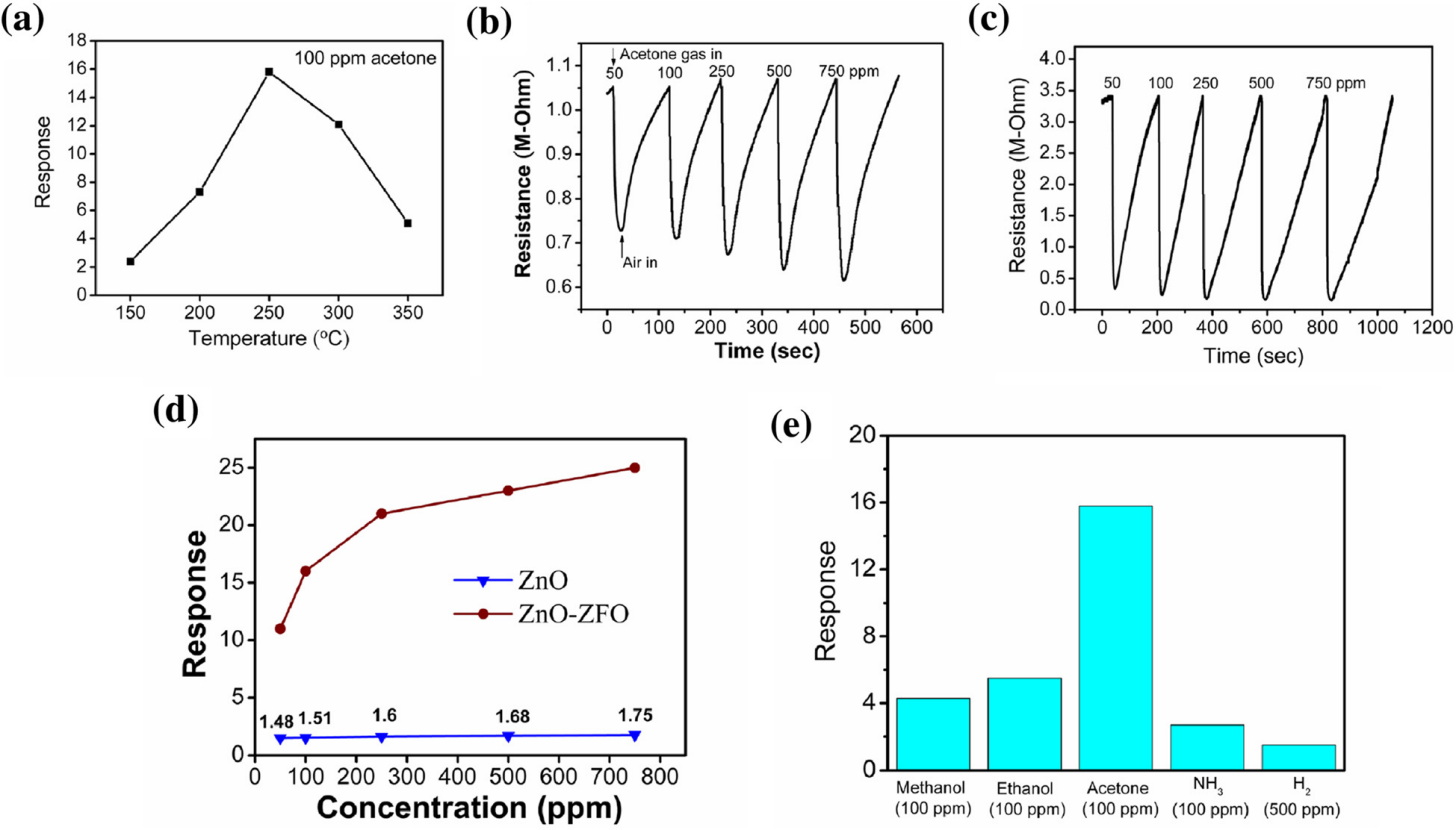
Gas-sensing properties of the ZnO–ZFO sensor: **a** gas-sensing responses vs. operating temperature of the ZnO–ZFO sensor upon exposure to 100 ppm acetone gas. Cyclic resistance change curves of the ZnO and ZnO–ZFO sensors to various acetone concentrations (50–750 ppm): **b** ZnO sensor **c** ZnO–ZFO sensor. **d** The summarized gas-sensing response values of the ZnO and ZnO–ZFO sensors upon exposure to various acetone gas concentrations. **e** The gas-sensing responses of the ZnO–ZFO sensor upon exposure to various reducing gases. The ZnO–ZFO sensor exhibited superior gas-sensing response to acetone gas in this work

We further investigated the acetone gas-sensing performance of the sensor composed of ZnO–ZFO nanostructures (referred to as ZnO–ZFO sensor hereafter). Notably, the gas-sensing response of metal oxide sensors results from the sensor resistance change on exposure to test gases. A proper operating temperature of the sensors is necessary to achieve a balance between the interaction of test gas molecules on the surface of the oxide and the test gas diffusion rates during the gas-sensing test, and to obtain an optimal gas response. The operating temperature-dependent gas-sensing response of the ZnO–ZFO sensor was conducted by exposing the sensor to 100 ppm acetone gas at a temperature range of 150–350 °C. The gas-sensing response of the ZnO–ZFO sensor initially increases with an increase in operating temperature and shows a maximum response of approximately 15.8 at 250 °C, followed by a decrease with a further increase of the sensor operating temperature (Fig. 3a). Figure 3b, c shows typical resistance change curves of the sensors composed of ZnO and ZnO–ZFO nanostructures on exposure to various acetone vapor concentrations at 250 °C. The cyclic sensor resistance change curves of the ZnO and ZnO–ZFO sensors in this study can be described as a decrease in the sensor resistance on exposure to acetone gas and complete recovery to the initial state on the removal of acetone gas. This is attributable to the characteristics of the n-type oxide semiconductors [19, 20]. The gas-sensing responses of the ZnO and ZnO–ZFO sensors were evaluated according to the resistance change curves, as shown in Fig. 3d. The gas-sensing response of the sensors increased as the acetone gas concentration was increased. The response of the ZnO–ZFO sensor to various acetone concentrations was higher than that of the ZnO sensor. The gas-sensing response of the ZnO–ZFO sensor increased from approximately 11 to 25 with an increase in the acetone concentration from 50 to 750 ppm. By contrast, the gas-sensing response of the ZnO sensor increased from 1.5 to 1.8 when the acetone concentration was increased from 50 to 750 ppm. The gas-sensing response of the ZnO nanostructures increased considerably by coating ZFO crystallites on the surfaces of the ZnO nanostructures. The depletion region plays a crucial role in carrier transportation in semiconductor oxides. The depletion layer thickness of semiconductor oxide nanostructures is approximately 20 nm at 300 °C [21]. In the current study, the sizes of the ZFO crystallites covering the ZnO core were in the approximate range of 10–20 nm. Presumably, most of the ZFO shell layers became fully depleted because the irregular surface morphology of ZFO crystallites enhanced their oxygen adsorption efficiency [22, 23]. Moreover, initially, a depletion region was formed at the ZnO/ZFO interface because of the difference in the work function between ZnO and ZFO [24]. An offset between the conduction bands of ZnO and ZFO in the ZnO–ZFO heterostructure has recently been reported [25, 26]. The high-efficiency surface adsorption of oxygen species with the interfacial depletion layer narrows the conducting channel of ZnO–ZFO heterostructures; a ZnO–ZFO sensor therefore has high resistance. These features might account for the potential barriers in the ZnO–ZFO system having a higher modulation size than those in pure ZnO on exposure to acetone gas [27]. Furthermore, the response and recovery times were calculated according to dynamic resistance change curves; they were defined as the durations of a 90 % change in the resistance amplitude on exposure to a test gas and air, respectively [16]. The response times of the ZnO sensor were approximately in the range of 11–18 s on exposure to acetone concentrations of 50–750 ppm. Moreover, the response time of the ZnO–ZFO sensor increased from 7 to 13 s when the acetone concentration increased from 50 to 500 ppm. The response times of the ZnO–ZFO sensor for various acetone concentrations were slightly shorter than those of the ZnO sensor under the same test conditions. The recovery times of the ZnO and ZnO–ZFO sensors were longer than the response times. The recovery time of the ZnO sensor increased from 41 to 52 s when the acetone concentration was increased from 50 to 500 ppm. By contrast, the recovery time of the ZnO–ZFO sensor was approximately 58 and 96 s on exposure to 50 and 500 ppm acetone, respectively. The response times of the sensors were considerably shorter than the recovery times. This is attributable to a reaction between the reducing gas molecules and adsorbed negative oxygen species occurring more easily than the oxygen to overcome the barrier to repopulating the vacant states of the oxide surfaces after desorption of oxygen on exposure to a reducing gas ambient [28]. Several oxide heterostructures have been found to show a recovery time longer than the response time in cyclic reducing gas-sensing tests [17, 29]; this difference is associated with the high surface-to-volume ratio of nanostructures. In the current study, a larger surface area and the presence of heterointerfacial contact in the ZnO–ZFO heterostructures resulted in a higher acetone gas-sensing response and a shorter response time compared with those of pure ZnO; the recovery times differed only slightly. Moreover, the ZnO–ZFO sensor was further exposed to various reducing gases at 250 °C for determining the performance of reducing gas-sensing selectivity of the ZnO–ZFO sensor. Figure 3e displays the gas-sensing responses of the ZnO–ZFO sensor on exposure to various reducing gases. The ZnO–ZFO sensor exhibited a distinct and superior gas-sensing response to acetone gas over the other reducing gases. The ZnO–ZFO sensor exhibited satisfactory gas-sensing selectivity in acetone gas among various reducing gases.

**Fig. 4:**
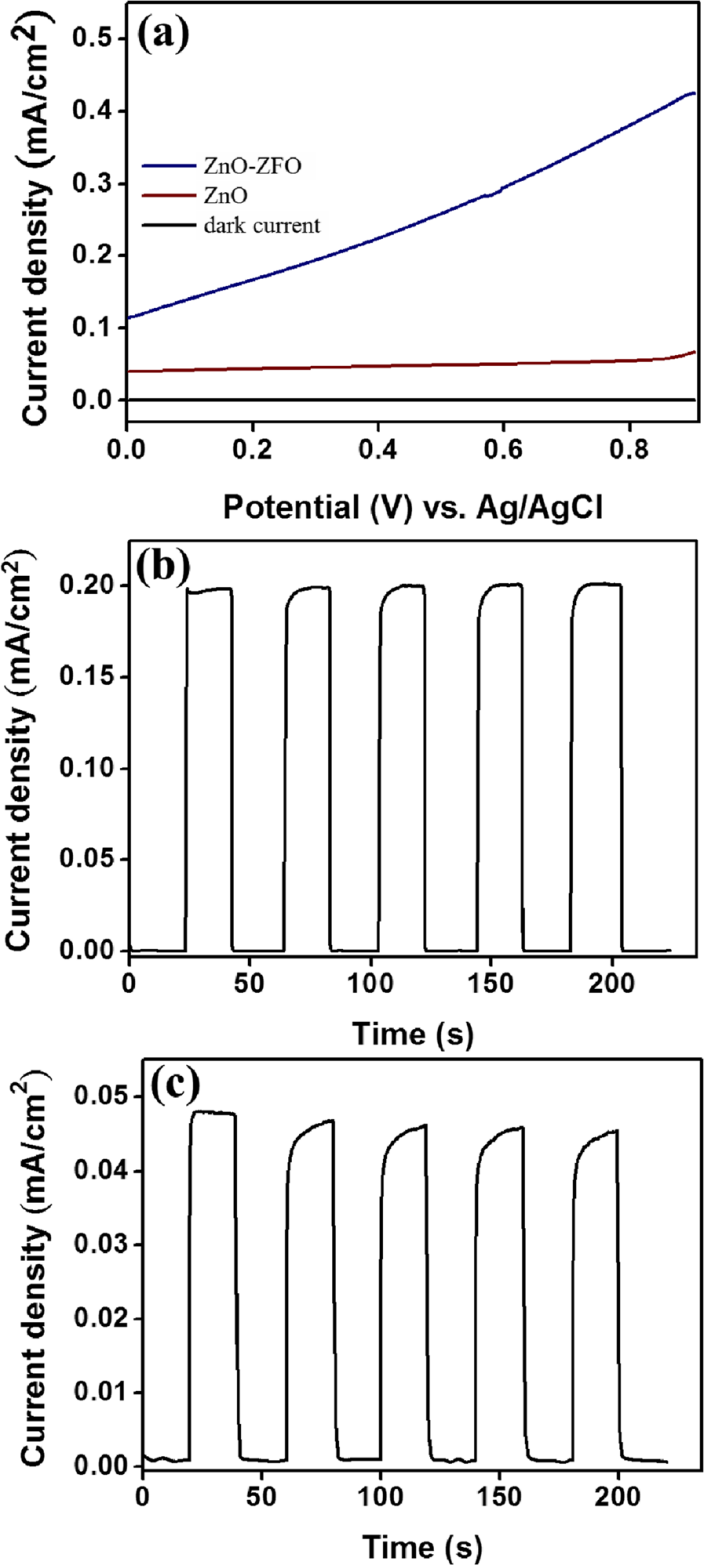
PEC characterization of the ZnO–ZFO: **a** the photocurrent density vs. potential curves of the ZnO and ZnO–ZFO. **b** Cyclic photocurrent density vs. time curves of the ZnO–ZFO at 0.3 V. **c** Cyclic photocurrent density vs. time curves of the ZnO at 0.3 V

The PEC activity of ZnO and ZnO–ZFO heterostructures was evaluated in Na_2_SO_4_ electrolyte. For investigating the photosensitivity of the prepared samples, the generated photocurrent was measured as a function of the applied potential under both light and dark conditions (from 0 to 0.9 V). Figure 4a shows current density–potential curves for ZnO and ZnO–ZFO in the presence and absence of illumination. Clearly, the dark current of both ZnO and ZnO–ZFO devices is low and negligible over the swept voltage ranges (in the order of 10^−4^ mA/cm^2^). Upon exposure to simulated solar light, ZnO–ZFO exhibited a higher photocurrent density compared with ZnO at the same applied potential. This result further confirmed the crucial role of ZFO crystallites in enhancing the photoactivity of ZnO–ZFO heterostructures. An amperometric study of ZnO and ZnO–ZFO heterostructures was performed at a fixed bias of 0.3 V and with on/off illumination cycles. The cyclic current density–time curves of the samples are displayed in Fig. 4b, c. The illuminating time was 20 s for each cycle. When the ZnO–ZFO electrode was illuminated by simulated solar light, the current density of ZnO–ZFO increased sharply to a steady value of approximately 0.2 mA/cm^2^, indicating that a stable photoelectrolysis reaction occurred. When light illumination was sporadic, the photocurrent rapidly recovered to its initial stable value. PEC cyclic tests of the ZnO–ZFO heterostructures showed the photocurrent response to be quick and highly reproducible (Fig. 4b). No substantially transient photocurrent was observed under illumination, indicating the high crystal quality of ZFO prepared through RF sputtering. In a previous study, ZFO with poor crystal quality created deep energy levels in the bandgap and exhibited a substantially transient photocurrent during PEC tests under illumination [15]. By contrast, under illumination, the increased photocurrent of ZnO was lower than that of ZnO–ZFO. Moreover, for ZnO, the photocurrent density–time curves showed a more rounded corner and a slight decrease in the photocurrent density with an increase in the test cycle number, reflecting the deterioration of the PEC performance with time (Fig. 4c). The enhancement of the PEC performance of ZnO because of the coating by homogeneous nanoscale ZFO crystallites herein was comparable with that reported in a previous study, in which ZFO nanoparticles with sizes of 5–8 nm were decorated on ZnO nanostructures through thermal decomposition of metal–surfactant complexes [15]. For n-type ZnO, on exposure to simulated solar light, the photogenerated electrons move to the F-doped SnO_2_ substrate, and the holes in the valence band move to the ZnO/electrolyte interface and oxidize the water at the interface. By contrast, in ZnO–ZFO heterostructures, the coating of ZFO crystallites on the ZnO nanostructures to form a serrated surface extends the absorption range to the visible light region considerably and enhances the electron–hole separation efficiency, which facilitates highly efficient charge transfer to the electrode/electrolyte interface [24]. Similar enhanced PEC behavior resulting from the surface effect and the presence of heterojunctions has been reported in other oxide heterostructures [15, 30]. Thus, ZnO–ZFO demonstrated relatively high PEC performance in the current study.

## Conclusions

Approaches involving thermal evaporation and RF sputtering were developed for the synthesis of heterostructures consisting of a crystalline ZFO-aggregated shell layer and core ZnO nanostructures. When such ZnO–ZFO heterostructures were used as acetone-sensing materials, the gas sensor fabricated from the ZnO–ZFO heterostructures showed considerably higher acetone gas-sensing response in comparison with the sensor fabricated from pure ZnO nanostructures. The markedly enhanced acetone gas-sensing response is ascribed to the large surface area of the heterostructures and the formation of heterojunctions between ZnO and ZFO. The ZnO–ZFO heterostructures exhibited high photocurrent response under sunlight illumination during PEC tests. The increased area of the rugged surfaces of the heterostructures and the relatively small bandgap of the ZFO crystallites explain the superior PEC performance of the heterostructures compared with the PEC performance of pure ZnO.
